# Gender‐ and age‐specific association of known type 2 diabetes mellitus on incident mild cognitive impairment five years later: Results from the population‐based Heinz Nixdorf Recall Study

**DOI:** 10.1002/alz.084652

**Published:** 2025-01-09

**Authors:** Martha Jokisch, Anna Lena Platzbecker, Janine Gronewold, Karl‐Heinz Jöckel, Susanne Moebus, Raimund Erbel, Sara Schramm, the Heinz Nixdorf Recall Study Investigative Group

**Affiliations:** ^1^ University Hospital Essen, University of Duisburg‐Essen, Essen Germany

## Abstract

**Background:**

Type 2 diabetes mellitus (T2DM) is considered a risk factor for dementia. The association of T2DM and mild cognitive impairment (MCI) is reported inconsistently and appears to be gender‐ and age‐specific. The aim of the present study was to investigate the (gender‐ and age‐specific) risk of incident MCI five years later in initially cognitively healthy participants with vs. without known T2DM.

**Method:**

1467 subjects of the second (t1) and third (t2) follow‐up examination of the prospective, population‐based Heinz Nixdorf Recall Study (t1: 2005‐2009, Ø62.9 years; t2: 2010‐2015, Ø68.1 years) with complete data regarding T2DM (t1), complete cognitive assessments (t1&t2) and with a cognitive performance within the age‐ and education‐adjusted norm (cognitively healthy; t1) were included. MCI at t2 was defined present, if the cognitive performance was below the age‐ and education‐adjusted norm in at least one cognitive domain with or without reported subjective cognitive decline. Known T2DM (t1) was defined as self‐reported physician‐confirmed diagnosis or use of antidiabetic medication (n = 145 (9.8%)). Using log‐linear regression analyses with Poisson distribution, the relative risk (RR) for incident MCI with 95% confidence interval (CI) for T2DM vs. no T2DM was calculated (adjusted (adj.) for age, body mass index, smoking status according to directed acyclic graph; stratified by gender and age (50‐65 years, middle‐aged & 66‐80 years, old‐aged)).

**Result:**

Overall, 57 (39.3%) participants with T2DM vs. 363 (27.5%) without T2DM met criteria for MCI at t2. There was an increased RR of incident MCI (adj.: RR 1.31, 95% CI 0.98‐1.74) in participants with T2DM at t1. After stratification, the association was primarily present in middle‐aged participants (adj.: RR 1.67, 95% CI 1.09‐2.56 vs. RR 1.09, 95% CI 0.74.‐1.60 in old‐aged participants), especially in middle‐aged men (adj.: RR 1.84, 95% CI 1.09‐3.11 vs. RR 1.29, 95% CI 0.59‐2.83 in middle‐aged women).

**Conclusion:**

Our longitudinal data confirm T2DM as a risk factor for incident MCI, especially for men aged 50‐65 years. Prevention of T2DM therefore could help to preserve cognitive performance and delay the development of dementia.

